# Widespread service fragmentation for patients and families with tuberous sclerosis complex (TSC) in the Republic of Ireland

**DOI:** 10.1007/s44162-024-00049-8

**Published:** 2024-08-19

**Authors:** M. Vasseghi, C. Behan, A. Connolly, D. Cunningham, E. Dempsey, C. Flynn, M. Galvin, G. Griffin, P. Moloney, M. Murphy, Y. Owen, S. O’Malley, G. O’Rourke, O. O’Sullivan, C. P. Doherty

**Affiliations:** 1 Academic Unit of Neurology, School of Medicine, Trinity College Dublin (The University of Dublin), Dublin, Ireland; 2 St James’s Hospital, Dublin, Ireland; 3 Childrens Health Ireland at Tallaght, Dublin, Ireland; 4 Mater Misericordiae Hospital, Dublin, Ireland; 5 St. Vincent’s University Hospital, Dublin, Ireland; 6 Childrens Health Ireland, Dublin, Ireland; 7 Beaumont Hospital, Dublin, Ireland; 8 Cork University Hospital, Cork, Ireland; 9 Childrens Health Ireland at Temple Street, Dublin, Ireland; 10 Sligo University Hospital, Sligo, Ireland; 11 University Hospital Limerick, Limerick, Ireland; 12 FutureNeuro, SFI Research Centre, Dublin, Ireland

**Keywords:** Tuberous sclerosis complex (TSC), Audit, Care, Recommendations, Coordination

## Abstract

**Background:**

Tuberous sclerosis complex (TSC) is a rare approximate 1:6000 birth incidence, a genetic disease with a wide variability of physical and neuropsychiatric symptoms. Patients require lifelong care from multiple healthcare specialities, for which International and United Kingdom (UK) TSC consensus recommendations exist. Personalised care delivered by a centralised coordinated team of TSC experts is recommended. There is no such service for the estimated 600 TSC patients in the Republic of Ireland (ROI) and there is a paucity of information regarding the healthcare of this group.

**Purpose:**

Evaluate the baseline care of patients with TSC attending epilepsy services in the Republic of Ireland (ROI) against UK TSC consensus recommendations.

**Methods:**

Patients with a diagnosis of TSC attending 12 adult and paediatric epilepsy centres in the ROI were identified. Clinical audits measured the baseline care of a subset of these patients against UK, TSC clinical recommendations. Data was anonymised and analysed at Trinity College Dublin.

**Results:**

One hundred thirty-five TSC patients attending twelve epilepsy centres were identified. Adults (*n* = 67) paediatric (*n* = 68). The care of 83 patients was audited (*n* = 63 ≥ 18 years) and (*n* = 20 < 18 years). Many baseline tests were completed, however, they required intra or interhospital referral. Care appears fragmented and there was no evidence of formal disease surveillance plans.

**Conclusions:**

The number of TSC patients attending epilepsy services is lower than expected (*n* = 135). Specialist services and treatments for TSC are available through informal referral pathways. Although UK, TSC consensus baseline recommendations are roughly adhered to, care is fragmented. Increased coordination of care could benefit disease management.

**Supplementary Information:**

The online version contains supplementary material available at 10.1007/s44162-024-00049-8.

## Introduction

Tuberous sclerosis complex (TSC) is a rare autosomal dominant, multisystem genetic disorder, affecting approximately. 1:6000–1:10,000 live births [1, 2]. It is caused by a pathogenic variant in the TSC1 or TSC2 gene, resulting in the dysregulation of the mTOR pathway, and the growth of benign tumours (hamartomas) in numerous organ systems, including the brain, lungs, kidney, heart, eyes, oral cavity, endocrine and skeletal systems [3]. Significant manifestations include epilepsy 84%, renal complications 50%, pulmonary involvement (primarily in females) 80%, and associated neuropsychological disorders (TAND) 90%, which encompass psychiatric, intellectual, behavioural, neuropsychiatric, psychosocial and academic difficulties [4–8].

TSC occurs in both genders, and all ethnic groups and symptoms of TSC vary considerably from mild to severe, with symptoms emerging and changing throughout the person’s lifetime and may lead to death [9].

Considerable progress in the understanding of TSC has led to the development of novel targeted therapeutics with remarkable results. Mammalian target rapamycin (mTOR) inhibitors have emerged as the cornerstone of TSC tumour management, generating disease control and improved outcomes [3, 10, 11]. It is vital that individuals with TSC obtain an early diagnosis and receive optimal personalised care for their condition throughout their entire lifespan [3, 12].

International and United Kingdom (UK) TSC Consensus Clinical Recommendations define the best approach to diagnosis, surveillance and management [3, 7, 13]. Due to the multiorgan and complex nature of the disease, personalised care, ideally delivered through TSC clinics/hubs of expertise, is recommended [3, 14].

Based on an estimated international prevalence of 1:6000–1:10,000, there should be approximately 600 individuals with a diagnosis of TSC in the Republic of Ireland (ROI). These patients do not have access to a specialist TSC clinic/hub or formalised network of TSC expertise in the ROI, and there is a paucity of information concerning their identity, well-being, and the healthcare they are receiving. The aim of this study was to evaluate the baseline care of patients with TSC attending epilepsy services in The Republic of Ireland (ROI) against UK TSC consensus recommendations and inform service improvements. This coordinated care approach improves patient care and safety and is in line with the World Health Organisation (WHO) guidelines, where patient safety within healthcare systems is seen as a framework of organised activities creating cultures, environments, behaviour, processes and procedures that lower risks and reduce the occurrence of avoidable harm [15].

## Methods

Patients with a diagnosis of TSC attending eight adult and four paediatric epilepsy services in the Republic of Ireland (ROI) were identified through the Epilepsy Electronic Patient Record (EEPR), Fitzsimons et al. [16] and chart review. The UK TSC consensus recommendations were used to create an audit tool of 46 questions, which included patient characteristics, genetics, central nervous system, kidney, lung, heart, eyes, skin, liver, pancreas and access to specialists and treatments. Clinical audits measured the care of 83 of the 135 identified patients against this tool.

An initial audit of two adult epilepsy services *n* = 41, was described by Behan et al. [17] and a follow-up study extended the audit nationally to a further six adult and four paediatric services over a 6-month period in 2022. Ethical approval was obtained from Trinity College Dublin. Audit participation was granted at each site and data-sharing agreements were put in place. Patient and site identifiers were removed, and data was uploaded anonymously to a Qualtrics platform by the participating healthcare practitioners at each site. The final data set amalgamates the two audits and was analysed on IBM SPSS 28 at Trinity College Dublin.

The rational for conducting the audit in epilepsy services as opposed to within a different speciality, was based on a number of factors. Epilepsy occurs in approx. 84% of people with TSC [18]. A National Epilepsy Electronic Patient Record (EEPR), Fitzsimons et al. [16] is available across epilepsy services in the ROI and facilitated the execution of the audit. There is no national individual patient identifier or electronic health record system in the ROI and no registry of TSC patients.

## Results

One hundred thirty-five TSC patients attending twelve epilepsy centres were identified. The care of a subset of 83 patients was audited. This number was due to the work demands on staff subsequent to the COVID-19 pandemic and the difficulties using electronic information material due to a cyber-attack on the Health Service Executive (HSE) Information Technology (IT) systems.

**Fig. 1:**
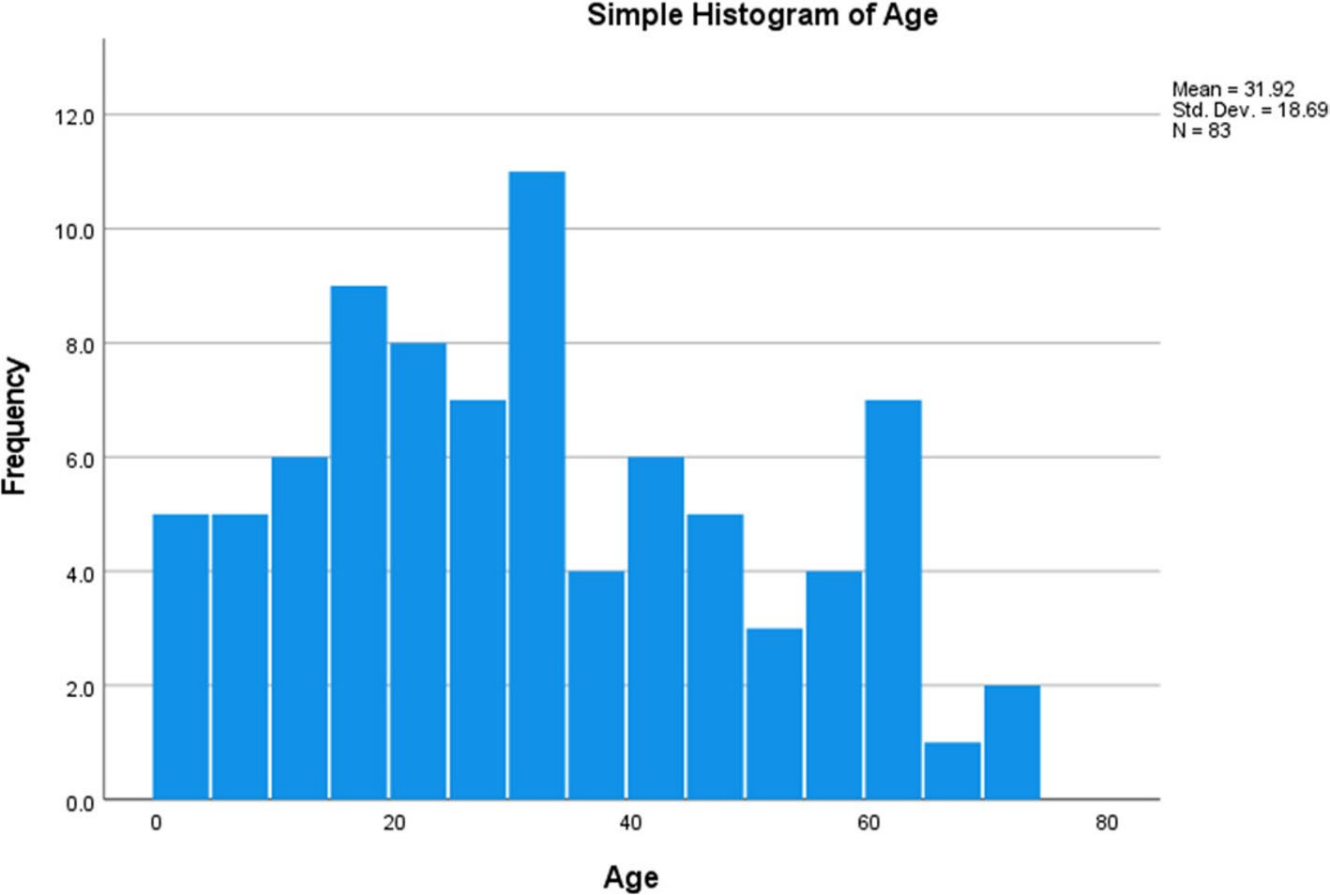
Age profile of audit participants

Demographic participant information is summarised in Table 1 and Fig. 1.

**Table 1:** Demographic characteristics of audit participants. Overall (*N* = 83)

Age categories	Gender	Age/mean/range/max/min	
Under age 18	Male	*N*	10
		Mean age	7.60
	Female	*N*	10
		Mean age	10.60
Age 18–75	Male	*N*	39
		Mean age	39.95
		Range	55
		Minimum	18
		Maximum	73
	Female	*N*	24
		Mean age	38.46
		Range	43
		Minimum	19
		Maximum	62

**Fig. 2:**
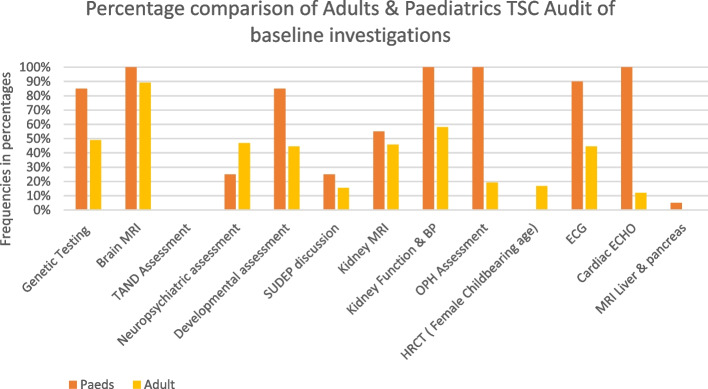
Comparing baseline investigations of adult and paediatric TSC patients, to that recommended by UK TSC consensus recommendations, in 12 epilepsy centres in the Republic of Ireland. *MRI* magnetic resonance imaging, *TAND* tuberous sclerosis neuropsychiatric disorder, *SUDEP* sudden unexpected death in epilepsy, *BP* blood pressure, *OPH* ophthalmology, *HRCT* high-resolution computerised tomography, *ECHO* echocardiogram, *ECG* electrocardiogram

The findings of baseline investigations are summarised in Fig. 2.

**Fig. 3:**
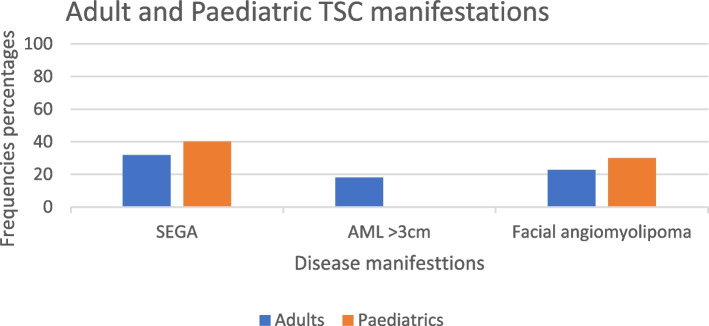
Subependymal giant cell astrocytoma (SEGA), renal angiomyolipoma (AML). Facial angiofibroma

The presence of three manifestations of TSC are summarised in Fig. 3.

Brain MRIs were carried out for 89% of adults and 100% of children.

Sudden unexpected death in epilepsy (SUDEP) was discussed in 15% of adults and 25% of children.

TAND (tuberous sclerosis-associated neuropsychiatric disorders) which affects 90% of patients were not assessed with the TAND assessment tool in any adult or paediatric services. However, other neuropsychological assessments were carried out in 47% of adults and 25% of paediatric cases as well as developmental status assessments in 44% of adult and 85% of children. Seventeen percent of females of childbearing age were documented as having had an HRCT and 5% of all patients had an MRI of the liver and pancreas.

MRI is the recommended diagnostic tool for renal imaging, but ultrasound was used in 13.3%, and computed tomography (CT) and ultrasound in 3.5%.

### Three TSC disease manifestations

The presence of subependymal giant cell astrocytoma (SEGA) was identified in 32% of adults and 40% of children. This is higher than the reported 6–25% in the literature [19] and may be due to selection bias given only patients from epilepsy services were audited. Both surgical treatment and the use of mTOR inhibitors were available, with everolimus the preferred first-line treatment option.

Renal angiomyolipoma (AML) > 3 cm was present in 18% of adults and none were reported in the paediatric group.

Facial angiofibroma was seen in 23% of adults and 30% of children and topical mTOR is being prescribed.

### Access to specialists and treatments

All specialities required for the care of TSC patients were available in the ROI. Including Neurology, Nephrology, Dermatology, Psychiatry, Psychology, Endocrinology, Genetics, Respiratory medicine and Interventional radiology. There is one advanced nurse practitioner (ANP) in Ireland with specific training in TSC but there are ANP epilepsy nurses in every centre audited.

Multidisciplinary teams for the care of patients with SEGA were available within hospitals for 45% of adults and 100% of paediatric cases. Surgery and the use of mTOR inhibitors for SEGA and epilepsy were available for 100% of all patients through referral pathways. MRI under general anaesthetic was also available; however, it was reported as often being difficult to organise. Video electroencephalogram (EEG) is also performed in designated national services. Access to interventional radiologists and child and adolescent mental health services CAHMS also required referral. Everolimus although off label is being prescribed for patients with SEGA, epilepsy, Renal AMLs and Lymphangioleiomyomatosis (LAM), and topical mTOR inhibitor is being used for facial angiofibroma. Regular Fundoscopy examination was reported in 75% of children and 5% of adults. 20% of children and 11% of adults were identified as having regular ECGs.

## Discussion

The main finding of this audit is that many baseline investigations were completed, especially in the paediatric services. However, care appears fragmented and separate referrals were required to access services and treatments even within the same hospital. Free text comments of participating staff indicated lengthy referral times, particularly to CAHMS and there was no centralised care coordination.

The epilepsy centres where the audit was conducted are staffed by highly qualified Advanced Nurse Practitioners (ANPs) in Epilepsy care, who deliver specialised care to their patients for all of their neurological issues. It is not considered their remit to be coordinators of the extensive multidisciplinary care needs of TSC patients. Subsequently, coordination of the many investigations and consultations required to manage TSC frequently falls on the shoulders of the patients, families and carers to navigate their care and healthcare system. This is not conducive to the International or UK TSC recommendations where lifelong, personalised, multidisciplinary care by a coordinated team of TSC experts is recommended. Nor is it in line with the WHO guidance on safe systems of care to improve patient safety and reduce avoidable harm [15]. The National Rare Diseases Office is developing rare disease care pathways, including one for TSC; however, it is not known when this will be completed [20].

The low numbers attending epilepsy services is unexpected (*n* = 135). Based on the frequency of epilepsy in TSC (84%) [18] and a prevalence of TSC 1:6000–1:10,000, an estimated 400 patients would be expected to attend epilepsy services. The epidemiological data would suggest that in excess of 600 patients should be attending some type of surveillance services for their disease. It is possible that a number of patients are attending neurologists elsewhere, e.g., privately or having their epilepsy managed by their general practitioner (GP) or paediatrician. However, given the high rate of refractory epilepsy in TSC the numbers are still less than expected. While the national epilepsy services use a national electronic health record, in general, patients with multisystem diseases like TSC are not catered for in a common record and the lack of this as well as the use of telehealth and other digital health and remote monitoring systems is a significant failure in the Irish health system [21].

Of note, is that over a 5-year period 2016–2020, Doody et al. [22] looked at the presenting problems of patients with an intellectual disability who were admitted to acute hospitals. TSC was the fifth most common condition and many of the categories of presenting problems were relevant to TSC. This begs the question of whether patients are having their care needs addressed in a proactive surveillance model or a reactive acute unscheduled care model.

Everolimus, an mTOR inhibitor, is approved in many countries for the treatment of renal angiomyolipoma, lymphangioleiomyomatosis (LAM) and/or partial-onset epilepsy associated with TSC in addition to SEGA [3, 11]. The European Medicines Agency (EMA) has granted a licence for its use in TSC in Europe [23]. However, a subsequent application for its use in the ROI has not been made and Everolimus is currently being prescribed off-label for the care of patients with TSC.

The audit was limited in that it captured the UK and International TSC baseline recommendations and not ongoing surveillance. Due to the COVID-19 pandemic and the Health Service Executive (HSE) cyber-attack, staff workload and difficulty in accessing files affected their capacity to audit all of the TSC patients attending their services. The absence of an electronic patient record system in Ireland also impacted the study.

## Conclusion

The number of TSC patients attending epilepsy services is lower than expected (*n* = 135). The UK, TSC consensus baseline recommendations are broadly adhered to. Specialist services and treatments for TSC are available, however, these require intra and inter-hospital and service referrals with no clear feedback system of their outcomes. TSC care is fragmented and lacks a formal TSC clinic or network to provide TSC care for this multiorgan complex disease as urged by international and UK TSC clinical recommendations. Coordinated care ideally in a centre of excellence would facilitate healthcare professionals with TSC expertise to provide lifelong personalised care to TSC patients and support to their HCP colleagues. There would be an amplification in TSC awareness, increased patient safety and outcomes, improved patient and provider satisfaction and a reduction in healthcare costs.

### Supplementary Information

Supplementary Material 1.

## Data Availability

The datasets generated during and/or analysed during the current study are not publicly available due to data protection issues but are available from the corresponding author on reasonable request.

## Abbreviations

AML: Renal angiomyolipoma
ANP: Advanced nurse practitioner
BP: Blood pressure
CAHMS: Child and Adolescent Mental Health Services
CT: Computed tomography
ECG: Electrocardiogram
ECHO: Echocardiogram
EEG: Electroencephalogram
EERR: Epilepsy Electronic Patient Record
EMA: European Medicines Agency
HSE: Health Service Executive
HRCT: High-resolution computerised tomography
IBM SPSS: International Business Machines Corporation Statistical Package for the Social Sciences
IT: Information Technology
LAM: Lymphangioleiomyomatosis
MRI: Magnetic resonance imaging
mTOR: Mammalian target of rapamycin
OPH: Ophthalmology
ROI: Republic of Ireland
SEGA: Subependymal giant cell astrocytoma
SUDEP: Sudden unexpected death in epilepsy
TAND: Tuberous Sclerosis Neuropsychiatric Disorder
TSC: Tuberous sclerosis complex
U.K: United Kingdom
WHO: World Health Organisation
