# Supplementary material for: Widespread service fragmentation for patients and families with tuberous sclerosis complex (TSC) in the Republic of Ireland
Source: J Rare Dis (Berlin). 2024 Aug 19;3(1):24. doi: 10.1007/s44162-024-00049-8 (PMC11330946; doi:10.1007/s44162-024-00049-8)
Supplement: Supplementary file 1 — Supplementary Material 1. [file 44162_2024_49_MOESM1_ESM.pdf]

Supplementary information. Audit questions based on UK TSC recommendations.

Q1	Age
Q2	Gender
Q3	Has genetic testing been carried out?
	**Central Nervous System**
Q4	Has a brain MRI been completed at baseline?
Q5	Do you have access to MRI under GA if required?
Q6	Does this patient have a known SEGA lesion?
Q7	Do you have access to an MDT to discuss SEGA cases? (The MDT should consist of at least a neuroradiologist, neurologist and neurosurgeon)
Q8	Do you have access to Everolimus for SEGA ?
Q9	What is your first line treatment for SEGA- Surgery or Everolimus?
Q10	Has this patient had annual TAND check list completed?
Q11	If the patient has a neuropsychiatric disorder, have they been seen by a neuropsychologist according to the NICE guideline?
Q12	Has the patient had a baseline neuropsychology assessment?
Q13	Has the patient had a developmental status assessment?
Q14	If the patient has epilepsy, has SUDEP been discussed?
Q15	Do you have access to an epilepsy surgery programme if required?
Q16	Do you have access to Everolimus for epilepsy?
Q17	Do you have access to video EEG if required ?
	**KIDNEY**
Q18	Has a Kidney MRI been performed at baseline?
Q19	Does the patient have AML >3cm
Q20	Do you have access to everolimus for AMLs?
Q21	Has the patient's BP and kidney function been checked at baseline?
	**LUNG**
Q22	Is the patient female of child-bearing age?
Q23	If the patient is female, have they had an HRCT at baseline?
Q24	If the patient is a symptomatic adult male (chest symptoms), have they had an HRCT?
Q25	If the patient had LAM at baseline, have they had a repeat HRCT 5-10 years after the baseline scan?
Q26	If the patient had LAM at baseline, have they had their pulmonary function test checked yearly?
Q27	If the patient has progressive LAM and deteriorating lung function, do you have access to mTOR inhibitors?
	**HEART**
Q28	Has the patient had a baseline ECG?
Q29	If the patient is a child, have they had an ECHO?
Q30	Has the patient been having regular ECGs?
Q31	If the patient is an adult and has cardiac symptoms, have they had an ECHO?
	**EYES**
Q32	Has the patient seen an ophthalmologist at baseline?
Q33	Has the patient had fundoscopy examination during each clinic appointment? Please check 2 previous clinic letters.
	**SKIN**
Q34	Does this patient have facial angiofibroma?
Q35	Do you have access to topical mTOR inhibitors?
	**LIVER & PANCREAS**
Q36	Has the patient had an MRI of the liver and pancreas?
	**ACCESS**
Q37	Do you have access to an adult neurologist in your clinic if required?
Q38	Do you have access to a paediatric neurologist in your clinic if required?
Q39	Do you have access to a nephrologist in your clinic ?
Q40	Do you have access to a respiratory physician in your clinic?
Q41	Do you have access to a geneticist in your clinic ?
Q42	Do you have a specialist nurse in your clinic?
Q43	Is your service funded by the HSE?
Q44	Do you have access to CAMHS for this patient ?
Q45	Do you have access to an interventional radiologist ?
Q46	Who leads your clinic?
	Free text
